# Kidney tonifying traditional Chinese medicine: Potential implications for the prevention and treatment of osteoporosis

**DOI:** 10.3389/fphar.2022.1063899

**Published:** 2023-01-09

**Authors:** Yan Duan, Yu-Ting Su, Jie Ren, Qun Zhou, Min Tang, Juan Li, Shun-Xiang Li

**Affiliations:** ^1^ Hunan Engineering Technology Research Center for Bioactive Substance Discovery of Chinese Medicine, School of Pharmacy, Hunan University of Chinese Medicine, Changsha, China; ^2^ Hunan Province Sino-US International Joint Research Center for Therapeutic Drugs of Senile Degenerative Diseases, Changsha, China

**Keywords:** osteoporosis, kidney tonifying traditional Chinese medicine, pharmacological mechanism, active ingredients, safety and toxicity

## Abstract

The aging global population is increasingly affected by osteoporosis (OP), which is one of the most significant threats to the elderly. Moreover, its prevention and treatment situations have become increasingly severe. Therefore, it is imperative to develop alternatives or complementary drugs for preventing and treating osteoporosis. Kidney tonifying traditional Chinese medicine (KTTCM) has been used for the treatment of osteoporosis for a long time. Pharmacological studies have shown that kidney tonifying traditional Chinese medicine can promote osteoblasts, inhibit osteoclasts, and regulate the level of estrogen and plays vital roles in stimulating osteogenesis, restraining adipogenesis of marrow mesenchymal stem cells (MSCs), regulating the metabolism of calcium and phosphorus, and inhibiting oxidative stress. These effects are mediated by OPG/RANKL/RANK, BMP/Smads, MAPKs, and Wnt/β-catenin systems. To develop a safe, synergistic, effective, and homogenized TCM formula with robust scientific evidence to provide faster and more economical alternatives, the anti-osteoporosis ingredients and pharmacological mechanisms of kidney tonifying traditional Chinese medicine are recapitulated from the perspective of molecular and cell biology, and the safety and toxicity of kidney tonifying traditional Chinese medicine have also been reviewed in this paper.

## Introduction

Osteoporosis, a chronic metabolic bone disease, affects the distribution of bone microstructure and leads to a decrease in bone mass, leading to decreased bone strength and higher fracture risk. The occurrence and progression of OP are complex and influenced by multiple factors, such as genetics, environmental factors, and immune abnormalities (Barnsley et al., 2021). OP has gradually become one of the high-risk diseases, which not only severely impacts the quality of life of the elderly but also generates excessive financial and psychological burdens for the family and society. With the rapid growth of aging populations on a global scale, osteoporotic fractures have become a crucial public health concern worldwide (Clynes et al., 2020).

In therapeutic practice, several types of drugs are used for treating OP, such as bisphosphonates, estrogen, parathyroid hormones, and calcitonin. Nevertheless, they may cause many serious side effects, including gastrointestinal adverse effects, joint pain, and leg cramps related to bisphosphonates in the treatment of osteoporosis (Cheng et al., 2020). In Chinese medicine theory, “Zang-Fu” is completely different from that of the organ in western medicine; it includes a large group of organs with interrelated physiological functions, which suggests that its function is not restricted by a single organ. Therefore, the “kidney” in TCM has a wide range of physiological functions, which refers to the organ system related to reproduction, growth and development, digestion, endocrine metabolism, and other functions (Lin et al., 2018). “Kidney deficiency” is also a broad concept that comprises abnormalities of many systems, such as the urinary system, reproductive system, endocrine system, neuropsychiatric system, and digestive system (Wang et al., 2016). Kidney tonifying traditional Chinese medicine (KTTCM) refers to TCM formulas, botanical drugs, and chemical constituents that can treat deficiency of the kidney and restore physical function (He et al., 2017). The traditional Chinese medicine (TCM) theory indicated that spleen and kidney deficiency is an important reason for the etiology and pathogenesis of OP; in other words, fasciae and the loss of bone nourishment are the main disease mechanism for kidney deficiency. Furthermore, it can be cured by KTTCM under the theory of ‘kidney dominates bone' (Xue C et al., 2022). KTTCM plays essential roles in facilitating bone formation, inhibiting bone resorption, regulating bone remodeling balance, promoting sex hormone secretion, suppressing oxidative stress, and regulating calcium and phosphorus metabolism (Peng et al., 2022).

In this review, the literature was searched with keywords including osteoblast, osteoclast, and osteoporosis from PubMed, Medline, Web of Science, ScienceDirect, CNKI, and VIP database from 2000 to 2022. Relevant data were collected from Chinese traditional books, Chinese Pharmacopoeia, and TCM formulas to identify the anti-osteoporosis effects and potential mechanisms of TCM formulas, botanical drugs, and chemical constituents, as well as their toxicity. Finally, the possibility of KTTCM as an alternative therapeutic option for osteoporosis was discussed proactively, and subsequent studies of KTTCM were prospected.

## The modern interpretation of osteoporosis

### Definition and classification of osteoporosis

As a result of OP, bone mass and density decrease, bone microstructure deteriorates, and bones become brittle. Bone mineral density (BMD) provided by dual X-ray absorptiometry (DXA), quantitative ultrasound (QUS), quantitative computed tomography (QCT), and high-resolution peripheral quantitative computed tomography (HR-pQCT) as a measurable and powerful index determining osteoporosis is the current “golden standard” for osteoporosis diagnosis (Xia W et al., 2019). The World Health Organization (WHO) defines osteoporosis as a BMD of two and a half SD or less below population-specific peak bone mass (PBM), where PBM is a period of stable growth and accumulation of bone mass and subsequent bone loss post-BMD (Black and Rosen, 2016). In addition, biochemical markers of bone turnover, including bone formation markers and bone resorption markers, are also an important basis for the clinical diagnosis of osteoporosis, such as alkaline phosphatase (ALP), osteocalcin, tartrate-resistant acid phosphatase (TRACP), and serum C-terminal telopeptide of type 1 collagen (S-CTX). Currently, there are some commonly used drugs for treating OP, including bisphosphonate class, receptor activator of nuclear factor-κB ligand inhibitor (RANKL inhibitor), hormone replacement therapy (HRT), selective estrogen receptor modulator (SERM), recombinant human PTH, abaloparatide and romosozumab-aqqg (Markham, 2019). OP is considered to be the most common chronic metabolic bone disease in humans and can be divided into two major groups: primary osteoporosis and secondary osteodysplasia. Primary osteoporosis includes postmenopausal osteoporosis, senile osteoporosis, and idiopathic osteoporosis. Postmenopausal osteoporosis generally occurs in women within 5–10 years after menopause; senile osteoporosis generally occurs in the elderly after the age of 70; the cause of idiopathic osteoporosis is still unclear. Secondary osteoporosis is mainly caused by definite causes that affect bone metabolism, including diabetes, hyperthyroidism, blood system diseases, kidney diseases, and metabolic bone diseases, as well as antiepileptic drugs, glucocorticoids, and other drugs.

### Pathogenesis of osteoporosis

**FIGURE 1:**
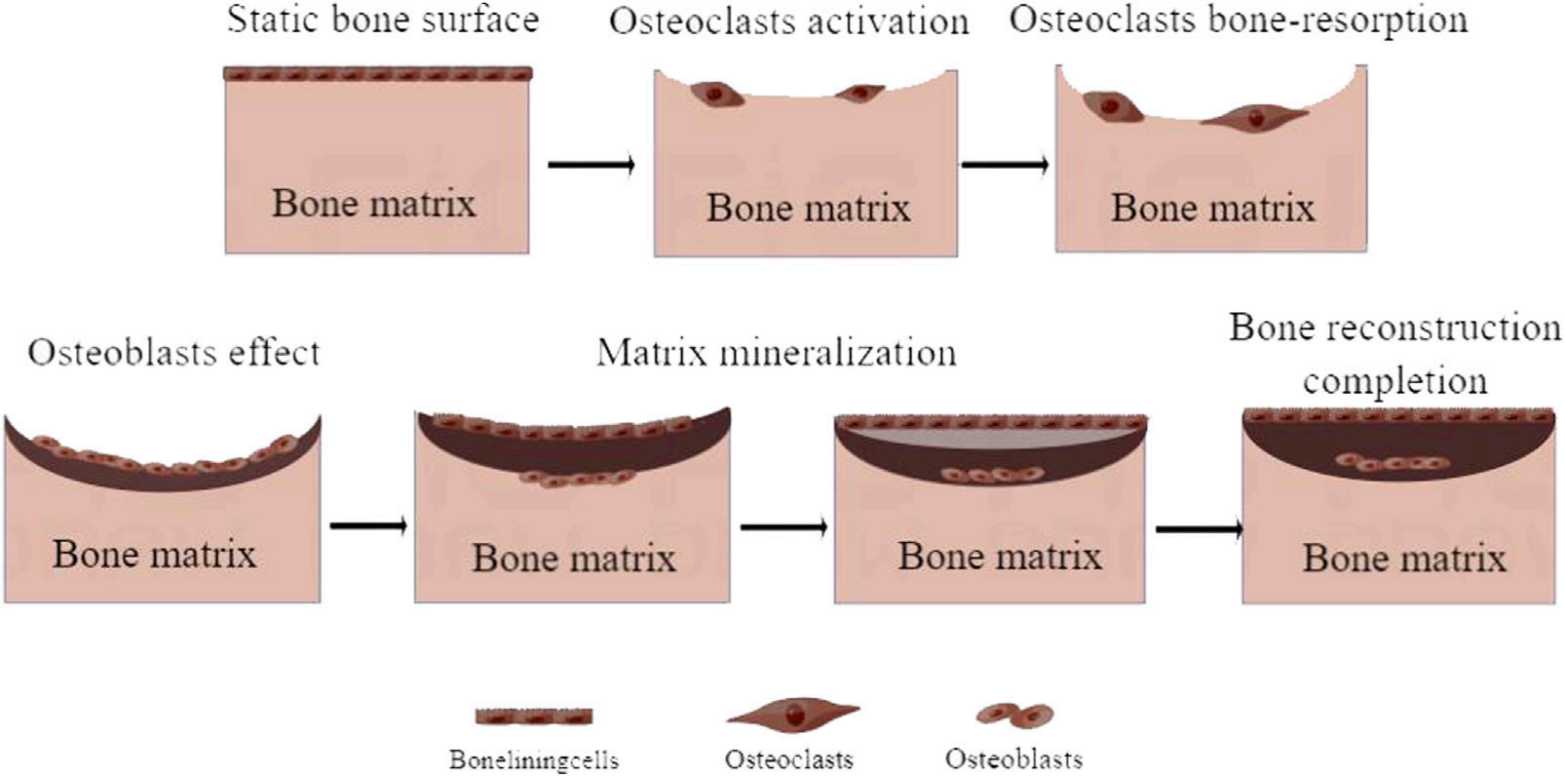
Process of bone remodeling.

Keeping the balance between osteoclasts and osteoblasts (bone forming and resorbing) is essential for maintaining bone homeostasis. This process, known as bone remodeling, comprises five phases of initiation, activation, resorption, formation, and mineralization (Figure 1), which are critical for bone shape and integrity. During the bone remodelling cycle, old or damaged bones are removed by osteoclasts (Yu et al., 2021). Osteoclasts (OC) perform bone resorption functions in bone remodeling, while osteoblasts (OB) are the main functional cells for bone formation (Borciani et al., 2020). Dysregulation of bone remodeling caused by various factors and complex mechanisms is the main reason for OP. If bone resorption is faster than bone formation, bone loss occurs, bone mineral content and bone matrix components decrease, cortical bone thins, and trabecular bone decreases, finally resulting in OP (Zhao et al., 2021).

The RANKL binds to RANK on OC precursors, which promotes OC differentiation. The proliferation and survival of OC depend on the binding of osteoblast-derived macrophage colony-stimulating factor (M-CSF) to the OC receptor c-fms. Interestingly, osteoprotegerin (OPG) secreted by OB can competitively inhibit the binding of RANK and RANKL; therefore, the ratio of RANKL/OPG is the key to determining bone resorption (Ono et al., 2020). In addition, decreased sex hormone levels, reduced function of the growth hormone–insulin-like growth factor (GH–IGF) axis, increased oxidative stress and glycosylation, sarcopenia, and reduced physical activity can also produce increased bone resorption, leading to lower bone strength (Schulman et al., 2011).

**FIGURE 2:**
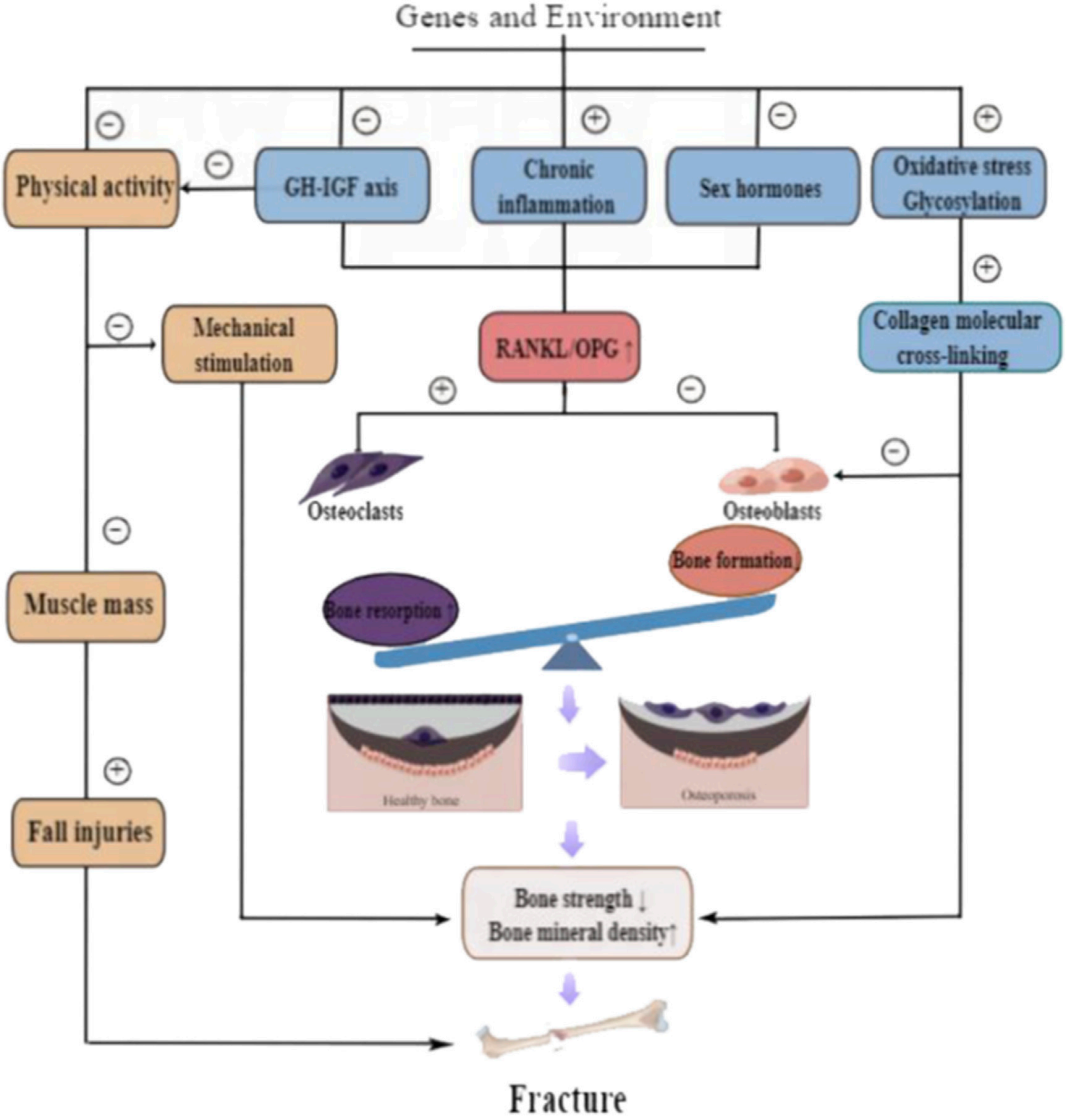
Pathogenesis of osteoporosis.

The occurrence of OP is the result of a complex combination of genetic and environmental factors, including aging, chemical drugs, estrogen deficiency, and decreased mechanical usage, which mainly affect bone strength, quality, structure, and internal properties. At the cellular level, it stimulates OC formation and osteocyte apoptosis. At the molecular level, it is mainly manifested in the regulation of the RANKL/OPG ratio, GH–IGF axis function, and oxidative stress (Figure 2).

## The understanding of osteoporosis from traditional Chinese medicine theory

Although there is no disease by the name of osteoporosis in traditional Chinese medicine, according to its clinical manifestations and pathogenesis, it belongs to the category of “bone Withers.” Su Wen (Plain Questions) is a classical text of ancient China, which claims that the basic pathology of “bone Withers” is “bone blight and bone marrow reduction.” Specifically, “bone blight” is equivalent to the degeneration and change of bone tissue microstructure, which is manifested by the thinning and fracture of bone trabecula, thus changing the normal load function of bone tissue and increasing bone fragility; “bone marrow reduction” corresponds to bone mass reduction, including bone matrix and bone minerals in equal proportion (Jin and Zheng, 2007). The “bone Withers” is used as a TCM clinical diagnosis of OP because the content of “bone Withers” is the same as the pathogenesis and clinical manifestation of OP. The theory of TCM holds that the kidneys store essence, dominate bone, generate marrow, and are the origin of congenital constitution, which indicates the existence of an intrinsic physiological relationship between the kidney, essence bone, and marrow and forms the theory of “kidney dominates bone.” The theory of TCM considers that the disease mechanism of OP is associated with the deficiency of kidney caused by factors such as aging, congenital inheritance, exertional injury, dietary habits, and mechanical stress (Ge et al., 2020). Modern studies of TCM have found that the incidence of kidney deficiency syndrome was progressively higher with increasing age, and bone mineral content in human bones also gradually decreases. In addition, hypofunction of the hypothalamic–pituitary–gonadal axis, with decreased levels of sex hormones, can cause decreased osteogenic function, making bone tissue content less per unit volume (Du and Li, 2022).

An intricate relationship exists between the kidney and bone; its parenchyma is that the kidney and its associated organs regulate bone formation and bone metabolism by secreting various hormones and cytokines. The kidney primarily influences the progress of osteoporosis by regulating the secretion of cytokines such as calcium and phosphorus, EPO, and 1α-hydroxylase.

The kidneys regulate two processes, namely, bone development and bone remodeling, through the excretion and reabsorption of calcium and phosphorus, and dysfunctional kidneys resulting in disturbed calcium and phosphorus metabolism will lead to osteoporosis.

When calcium-sensitive receptor (CaRare) is knocked out in mouse osteoblasts, their osteoblast differentiation ability is decreased, resulting in delayed bone development (Goltzman and Hendy, 2015). It has been found that calcium can induce osteoclast apoptosis by binding to CaRare on osteoclasts (Mentaverri et al., 2006). In addition, the elevated serum phosphorus caused by chronic renal insufficiency can also inhibit osteoclast differentiation and production (Bouillon et al., 2013).

Kidneys are the major organ generating the erythropoietin (EPO) hormone. EPO can not only impulse bone marrow stem cells and promote their differentiation into osteoclasts but also promote osteoclastogenesis in bone marrow monocytes (Sun et al., 2021). Intraperitoneal injection of EPO can promote cell proliferation, accelerate the rate of osteogenesis and angiogenesis, and promote cartilage callus transition in mice with femoral segmental bone defect model, thus exerting a pro-fracture-healing effect (Holstein et al., 2011). However, chronic kidney disease (CKD) can lead to a substantial reduction of Epo production in kidney cells (Miyauchi et al., 2021), thus affecting bone formation.

The kidneys synthesize 1α-hydroxylase to secrete active vitamin D-1,25 [1,25-(OH)2D3], which binds to the specific vitamin D receptor (VDR) on osteoblasts and osteoclasts to maintain bone homeostasis by affecting bone metabolism (Kiecker et al., 2016). 1α-Hydroxylase knockout mice can occur in impaired 1,25-(OH)2D3 synthesis and develop skeletal abnormalities characteristic of bone growth retardation and rickets (Bertoldo et al., 2022). The research has revealed that after local injury to the kidney induced by radiation in rats, the activity of renal 1α-hydroxylase is reduced, and the hydroxylation of vitamin D is blocked, followed by disrupted bone metabolism and poor mineralization of the bone matrix (Zhu et al., 2003). Thus, 1α-hydroxylase plays an essential role in bone mineralization and maintenance of bone homeostasis.

The liver is the primary organ storing blood and controlling the growth of muscles. The essence and blood are the identical source, and muscles are complementary to bone. Under such a premise, the essence and blood can nourish the bone marrow, and the muscles can sustain the bones. This presents evidence of the synergistic effects of the liver and kidney in the regulation of bones. The comprehensive disease mechanisms can clarify that the onset of OP is simply kidney deficiency, while also being associated with liver deficiency, and prevents OP when using kidney replenishment as the entry point. The research on KTTCM may provide useful information for the clinical application of OP treatment because of their characteristics, including stable efficacy, fewer adverse effects, and cheap and abundant resources.

## Kidney tonifying traditional Chinese medicine in the treatment of osteoporosis

KTTCM can improve the balance between bone and adipogenic differentiation of BMSCs, promote the secretion of sex hormones and cytokines, curb cellular oxidative stress, and regulate calcium and phosphorus metabolism for the prevention and treatment of OP. In this review, the chemical constituents and proposed mechanisms for KTTCM have been summarized in Table 1. We mainly focused on seven different KTTCMs and discussed their chemical constituents and anti-osteoporotic activity.

**TABLE 1:** Chemical constitutes and potential mechanism for each KTTCM.

Botanical drugs	Chemical constituents	Potential anti-osteoporotic activity	Potential mechanisms	Reference
Drynaria fortunei (Kunze ex Mett.) J. Sm. [Polypodiaceae; Drynariae Rhizoma]	Kaempferol, luteolin, naringin, catechin, and cyclolaudenol	Kaempferol and naringin may promote BMSC osteogenic differentiation and ameliorate the development of osteoporosis	Naringin exerts protective effects in GIOP by the PI3K/AKT/mTOR pathway. Naringin promotes BMSC osteogenic differentiation to ameliorate osteoporosis development by targeting JAK2/STAT3 signalling	Liu M et al. (2021), Wang et al. (2022), and Ge and Zhou (2021)
Epimedium brevicornu Maxim. [Berberidaceae; Epimedii Folium]	Icariin, epimedin A, magnoflorine, cupressoside A, and icarisideⅡ	Icariin might promote bone differentiation, improve osteoblast vitality, and promote bone binding	Epimedin C could alleviate glucocorticoid-induced suppression of osteogenic differentiation by modulating the PI3K/AKT/Runx2 signaling pathway. Icariin alleviates osteoporosis through EphB4/Ephrin-B2 axis	Huang et al. (2020), Xu et al. (2022), and Chen F et al. (2022)
Dipsacus asper Wall. Ex Henry [Dipsacaceae; Dispaci Radix]	Asperosaponin Ⅵ, loganin, sweroside, dipsanoside H, and acanthoside D	Asperosaponin Ⅵ reduces the differentiation of mononuclear osteoclasts and enhances osteogenesis	Asperosaponin Ⅵ can suppress osteoclastogenesis by stimulating the SMADs, TGF-β1, VEGFA, and OPG/RANKL signaling pathways. Sweroside induces the formation of mineralized bone matrix by regulating BMP2/CBFA1-mediated molecules	Chen Q et al. (2022) and Choi et al. (2021)
*Psoralea* corylifolia L. [Fabaceae; Psoraleae Fructus]	Psoralen, ispsoralen, bakuchiol, coryfolin, corylifolinin, bavachin, and bavachalcone	Psoralen can increase the proliferation and viability of hBMSCs. Psoralen alleviates radiation-induced bone injury by rescuing skeletal stem cell stemness	Psoralen accelerates the osteogenic differentiation of hBMSCs by activating the TGF-β/Smad3 pathway. Psoralen alleviates bone injury through AKT-mediated upregulation of GSK-3β and NRF2. Isopsoralen regulates PPAR-γ/Wnt to inhibit oxidative stress in osteoporosis	Huang et al. (2021), Yin et al. (2022), and Wang et al. (2018)
Cuscuta chinensis Lam. [Convolvulaceae; Cuscutae Semen]	Quercetin, kaempferol, hyperoside, and polysaccharide	Cuscutae Semen polysaccharide may exert a protective role in bone by promoting bone formation and inhibiting bone resorption.	Hyperoside reduces the expression of RANKL, TRAF6, IκBα, NF-κB p65, OPG, and NFATc1	Yang et al. (2022) and Chen et al. (2018)
Eucommia ulmoides Oliv. [Eucommiaceae; Eucommiae Cortex]	5-(hydroxymethyl)-2-furaldehyde, geniposidic acid, (p)-syringaresinol, aucubin, liriodendrin, and geniposide	Aucubin and geniposide slow the development of osteoporosis by inhibiting osteoclast differentiation. Geniposide ameliorates endoplasmic reticulum stress and mitochondrial apoptosis in osteoblasts	Aucubin increases the expression of collagen I, OCN, OPN, osterix, and phosphorylated Akt and Smads in bone tissue. Geniposide activates the expression of NRF2 and alleviates ER stress in MC3T3-E1 cells	Zhang Y et al. (2021), Li Y et al. (2020), and Yaosheng et al. (2022)
Euodia rutaecarpa (Juss.) Benth [Rutaceae; Euodiae Fructus]	Rubiadin, monotropein, polysaccharide, 2-hydroxy-1-methoxy-anthraquinone, and 1,3,8-trihydroxy-2-methoxy-anthraquinone	Monotropein and rubiadin-1-methyl ether can prevent bone loss in glucocorticoid-induced osteoporosis	Monotropein attenuates oxidative stress *via* Akt/mTOR-mediated autophagy in osteoblast cells	Xia T et al. (2019) and Shi et al. (2020)
Curculigo orchioides Gaertn. [Amaryllidaceae; Curculiginis Rhizoma]	Curculigoside, curculigine A, and orcinol glucoside	Curculigoside attenuates oxidative stress and osteoclastogenesis	Curculigoside mitigates oxidative stress and osteoclastogenesis by activating Nrf2 and inhibiting the NF-κB pathway	Liu H et al. (2021)
Achyranthis *Achyranthes* bidentata Bl. [Amaranthaceae; Bidentatae Radix]	Polysaccharide, quercetin, achyranthoside E, chikusetsusaponin Ⅳa, momordin Ⅰb, ecdysterone, daucosterol, isocyasterone, 5-epicyasterone, sengosterone, and cyasterone	Ecdysterone suppresses osteoclast differentiation and bone resorption activity	Ecdysterone enhances the activity of alkaline phosphatase, upregulates the expression of RANKL, and increases the serum content of calcium, phosphorus and TRAP in rats	Yi et al. (2019) and Tang et al. (2018)
Cornus officinalis Sieb. Et Zucc. [Cornaceae; Corni Fructus]	Gallic acid, ursolic acid, morroniside, sweroside, and cornuside	Morroniside can promote the differentiation of osteoblast and inhibit the differentiation of osteoclast	Morroniside might inhibit TRAP activity and TRAP-stained multinucleated positive cells	Lee et al. (2021)
Cervi Cornus Colla	Cervi Cornus Colla polypeptides and Cervi Cornus Colla polysaccharides	Cervi Cornus Colla polypeptides have protective effects on OVX rats	Cervi Cornus Colla polypeptides can inhibit IL-1 and IL-6 by nVAP and promote mitosis	Zhang et al. (2013)
Polygonum multiflora Thunb. [Polygonaceae; Polygoni Multiflori Rhizoma]	2,3,5,4-tetrahydroxystilbene-2-O-β-D-glucoside, emodin, physcion, schizandrin, and tetrahydroxystilbene glucoside	Tetrahydroxystilbene glucoside can promote MC3T3-E1 cell proliferation and differentiation	Tetrahydroxystilbene glucoside regulates OPG/RANKL/M-CSF expression *via* the PI3K/Akt pathway. Schizandrin protects against OVX-induced bone loss by suppressing ROS *via* Nrf2	Fan et al. (2018) and (Ni et al., 2020)
Ligustrum lucidum Ait. [Oleaceae; Ligustri Lucidi Fructus]	Nuzhenide, oleuropein, oleanolic acid, palmitie specnuezhenide, and salidroside	Ligustri Lucidi Fructus increases BMD, improves bone microstructure, and promotes osteoblast proliferation	Oleanolic acid can inhibit RANKL-induced osteoclastogenesis *via* ERα/miR-503/RANK signaling pathway in RAW264.7 cells	Xie et al. (2019)
*Dioscorea* opposita Thunb. [Dioscoreaceae; Dioscoreae Rhizoma]	Saponins, diosgenin, sapogenins, starch, purine derivatives, mucilage, Chinese yam polysaccharides, allantoin, and dioscorin	Diosgenin promotes the proliferation and differentiation of MG-63 cells	Diosgenin can increase the expression of Ki67, PCNA, OPN, BGP, β-catenin, Runx2, and cyclinD1	Ge et al. (2021)
Lycium barbarum L. [Solanaceae; Lycii Fructus]	Betaine, zeaxanthin, rutin, physalein, and ascorbic acid	Polysaccharide can promote osteoblast differentiation	−	Wang et al. (2020)
Cistanche deserticola Y. C. Ma [Orobanchaceae; Cistanches Herba]	Verbascoside, echinacoside, acteoside, cistanoside C, geniposide, and ononin	Cistanches Herba aqueous extract enhances BMD, increases ALP activity, and decreases the levels of DPD, cathepsin K, TRAP, and MAD.	Cistanches Herba aqueous extract might downregulate the levels of TRAF6, RANKL, RANK, NF-κB, IKKβ, and NFAT2 and upregulate the PI3K, AKT, OPG, and c-Fos expressions. Total glycosides and polysaccharides of Cistanches Herba could decrease the expressions of RANKL and p-β-catenin and upregulate the expression of BMP-2, OCN, OPG, and p-GSK-3β	Bo et al. (2019) and Fujiang et al. (2021)
Eclipta prostrata L. [Asteraceae; Ecliptae Herba]	Wedelolactone, apigenin, eclalbasaponins, and luteolin	Ecliptae Herba can improve bone micro-structure, inhibit osteoclast, increase the number of osteoblasts, and regulate the dynamic balance of bone absorption and formation	Ecliptae Herba alters and bone condition is improved *via* bacterial feeding *in vivo*	Zhao et al. (2019)

### *Drynaria fortunei* (Kunze ex Mett.) J. Sm. [Polypodiaceae; Drynariae Rhizoma]

There are many uses for Drynariae Rhizoma, such as tonifying the kidney and strengthening bone, repairing the injury, and relieving pain. It controls OP by regulating multiple signaling pathways and has the functions of increasing bone production, stabilizing bone structure, enhancing OB proliferation and differentiation, and inhibiting OC. In addition, Drynariae Rhizoma can increase the body’s absorption of calcium, promote calcium transport into bone, strengthen bone mineralization, maintain the balance of bone remodeling; regulate the metabolic level of the hypothalamic-pituitary-gonadal axis, improve endocrine function, inhibit the synthesis and secretion of inflammatory factors in bone metabolism, and have a potential anti-osteoporosis effect (Zhao et al., 2020a; Li J et al., 2021). Flavonoids are the main components of Drynariae Rhizoma in the prevention and treatment of OP. For instance, Hu et al. investigated the preventive effects of total flavonoids of Drynariae Rhizoma (TFDR) combined with CaCO3 on bone loss caused by estrogen deficiency. The results showed that TFDR can increase OPG content, reduce the content of RANK and RANKL, improve the bone loss of OVX rats, and promote the repair of fractured trabecular bone in OVX rats (Hu et al., 2021). TFDR also improved serum reactive oxygen species in OVX rats, SOD, and GSH-Px, reduced MDA, TNF-α, IL-6, and IL-1β levels, upregulated the expression of Runt-related transcription factor 2 (Runx2), OPG, and BGP, and downregulated the expression of p-AKT, p-PI3K, and p-mTOR, indicating that TFDR has anti-osteoporosis effects by enhancing the antioxidant capacity of rats (Mu et al., 2021). Li et al. found that TFDR (100 and 200 μg·mL-1) can improve ALP activity, increase the number of both mineralized nodules and osteogenesis-related proteins, and promote the mineralization of bone grafts and the differentiation of OB by activating the Wnt/β-catenin signaling pathway (Li R et al., 2021).

### *Epimedium brevicornu* Maxim. [Berberidaceae; Epimedii Folium]

In addition to invigorating the kidney and strengthening yang, Epimedii Folium also dispels pathogenic wind and eliminates dampness. Modern pharmacological studies have shown that the main potency substances of Epimedii Folium are flavonoids, which can repair bone defects by promoting bone differentiation, improving osteoblast vitality, and promoting bone binding for the prevention and treatment effects of OP (Martiniakova et al., 2020). For example, icariin (2 × 10–5 M) can promote the osteogenic differentiation of BMSCs and enhance the link of bone formation by enhancing the activity of ALP and upregulating the expression of BSP II and Runx-2 (Xu L et al., 2021). By using rat calvarial osteoblast culture and rat bone growth models, Shi et al. found that icariin (10–7, 10–6, and 10–5 M) increased peak bone mass attained in pups by activating the cAMP/protein kinase A (PKA)/cAMP response element-binding protein (CREB) pathway, and promoted the maturation and mineralization of rat skull OB (Shi et al., 2017). Icariin (250 mg·kg-1·day-1) also can inhibit the mRNA expression of PPARγ, CCAAT/enhancer binding protein α (C/EBP), and fatty acid-binding protein (FABP4) in OVX rats model of osteoporosis and downregulate the expression of notch1 intracellular domain of Notch (N1ICD) and Jagged1 protein in bone tissues (Liu et al., 2017).

### *Dipsacus asper* Wall. ex Henry [Dipsacaceae; Dispaci Radix]

Dispaci Radix can nourish the liver and kidneys, strengthen muscles and bones, continue fractures, and stop uterine bleeding, and it can be used to strengthen bones and heal fractures. Its main active ingredient, asperosaponin VI, can influence the proliferation and differentiation of OB through Wnt, JNK, and MAPK signaling pathways, promote bone formation, and display anti-osteoporosis activity (Zhao et al., 2020b). Asperosaponin VI (20 mg·kg-1·day-1) can also reduce the levels of IL-1β and TNF-α by inhibiting RANKL-induced OC differentiation and function and downregulate TRACP, cathepsin K (CTSK), matrix metalloproteinase-9 (MMP-9), and integrin-3β expression to exert its anti-osteoclast activity *in vitro* and *in vivo* (Liu et al., 2019). Another example is sweroside (1.3 × 10–9 M), which can induce the protein expression of ALP, estrogen receptor-α (ER-α), and G protein-coupled receptor 30 (GPR30), stimulate the phosphorylation of p38 kinase (p-p38), and promote OB differentiation and mineralization of MC3T3-E1 cells (Wu et al., 2020). Niu et al. determined the effect of D. asper extract (DRE) on bone mineral density and bone microarchitecture by establishing a hindlimb unloading rat model. The results showed that DRE (500 mg·kg-1·day-1) can not only improve the skeletal indexes, including bone volume fraction, thickness, density, trabecular number, and tissue mineral content but also ameliorate the trabecular separation and structure model index in rats (Niu et al., 2015). Moreover, the polysaccharide (50 and 200 mg·kg-1·day-1) from D. asper can upregulate the expression of vascular endothelial growth factor (VEGF) and OPG in OVX rats, downregulating the expression of RANK and RANKL by activating the PI3K/Akt/eNOS signaling pathway to cure PMOP (Sun et al., 2019).

### *Psoralea corylifolia* L. [Fabaceae; Psoraleae Fructus]

Psoraleae Fructus has the function of tonifying the kidney and strengthening yang, concentrating essence, and reducing urine. Psoralen, as the predominant component of Psoraleae Fructus, is known as a phytoestrogen and is used as a remedy for OP. Research has demonstrated that psoralen (1.5 × 10–5 M) can spur the proliferation of hFOB1.19 cells, increase phosphorylated extracellular-signal-regulated kinase (ERK), p38 and JNK levels, stimulate osteocytes proliferation, and promote osteoblasts differentiation (Li et al., 2017). Cai et al. found that both the 75% ethanol-only Psoraleae Fructus extract, and 75% ethanol plus petroleum ether, ethyl acetate, n-butanol, or water Psoraleae Fructus extract can stimulate the proliferation of MG-63 cells, increase ALP function, upregulate the expression of ERα, ERβ, and β-Catenin, and have estrogen-like effects and anti-osteoporosis activities (Cai et al., 2021). Chai et al. have shown that psoralen (10–7 M) and bakuchiol (10–7 M) can significantly inhibit TRACP activity by blocking the activation of AKT and AP-1 pathways, reducing the number of OC and the area of bone lacunae, decreasing expression and nuclear translocation of phosphorylated c-jun, and inhibiting the increase in AKT phosphorylation in OC, thereby improving the differentiation of OC and bone resorption induced by M-CSF and RANKL (Chai et al., 2018). Zhang et al. found out that psoralen (4 × 10–5 M) can activate osteoblast activity through the ERK signaling pathway to promote the mRNA expression of osteoblast differentiation markers, such as Runx2, osterix (OSX), osteopontin (OPN), and bone sialoprotein (BSP), and induce osteogenic differentiation and enhance fracture healing (Zhang T et al., 2019).

### *Cuscuta chinensis* Lam. [Convolvulaceae; Cuscutae Semen]

With Cuscutae Semen, liver and kidney function are nourished, the essence can be consolidated, uterine bleeding can be stopped, eyesight can be strengthened, and it can be used for bone strength. Its main active ingredients are flavonoids, lignans, quinic acid, and polysaccharides, which have estrogen-like effects and can be used for the prevention and treatment of OP (Yang et al., 2011). Cuscutae Semen polysaccharide with a purity of 78.73% can promote bone formation and inhibit bone resorption in OXV rats, exert a protective role in bone by increasing BMD, IGF, transforming growth factor-β (TGF-β), OPG, and osteocalcin, reducing TRAP and CTX levels, upregulating the expression of osterix, BMP-2, Runx2, and Smad5, and downregulating the expression of TRAP, NFATc1, and c-Fos(Liu X et al., 2020). Cuscutae Semen 80% ethanol extract (2 and 4 g·kg-1) can reduce serum TRACP-5b and RANKL levels, increase OPG levels, and downregulate c-fos, c-Src kinase, and NFATC1 protein expressions in OVX rats, implying that C. Semen extract may act through the c-fos/c-Src kinase/NFATC1 signaling pathways to play the role of anti-osteoporosis (Yun et al., 2021). Guo et al. established an OVX model and investigated the therapeutic effect of Cuscutae Semen on OP. These results illustrated that Cuscutae Semen Flavonoids (100 mg·kg-1) could inhibit the apoptosis of OB by improving the bone mass index, BMD and serum calcium, ALP level, regulating the expression of Bax, Bcl-2, OPG, and other proteins in the femur (Guo, 2018).

### *Eucommia ulmoides* Oliv. [Eucommiaceae; Eucommiae Cortex]

Eucommiae Cortex can be used to invigorate the liver and kidney, strengthen muscles and bones, and promote tocolysis. It contains many chemical constituents, including lignans, iridoids, phenols, steroids, terpenes, and flavonoids (Bai et al., 2019). Zhang et al. established a rat model with hindlimb suspension to explore the effect of Eucommiae Cortex on disuse-induced osteoporosis. The result showed that Eucommiae Cortex extract (300 mg·kg-1·day-1) can effectively prevent bone loss caused by hindlimb suspension, suppress bone turnover markers and urinary calcium and phosphorus levels, enhance the biomechanical strength of bone, and prevent the deterioration of bone trabecular microstructure (Yalei et al., 2014). Simultaneously, geniposide (50 and 100 mg·kg-1·day-1) can effectively reverse the pathological changes of trabecular bone induced by dexamethasone in rats, downregulate the expression of ATF4/CHOP, relieve ER stress, and improve DEX-induced mitochondrial apoptosis in MC3T3-E1 cells (Yaosheng et al., 2022). Total lignans (40 mg·kg-1·day-1) from Eucommiae Cortex can significantly prevent OVX-induced decrease of maximum stress and young modulus, regulating the expression of OPG and RANKL to induced primary osteoblastic cell proliferation and differentiation (Zhang et al., 2014). 5-(hydroxymethyl)-2-furaldehyde (5-HMF), which was isolated from Eucommiae Cortex, can upregulate the mRNA expressions of ALP, Col I-α, OCN, and OPN in BMSCs, downregulate the mRNA expressions of PPARγ, FABP4, and C/EBPα in adipocytes, and enhance the formation of mineralized nodules in OB. This result revealed that 5-HMF (50, 100, and 200 μg·mL-1) was a commanding inhibitor of adipogenesis and enhancer of osteoblastogenesis (Tan et al., 2014).

### *Euodia rutaecarpa* (Juss.) Benth [Rutaceae; Euodiae Fructus]

Euodiae Fructus can nourish the liver and kidney, strengthen muscles and bones, and dispel rheumatism, mainly including compounds such as polysaccharides, anthraquinones, flavonoids, and iridoids (Zhou et al., 2021). The crude polysaccharide MO90 isolated from Euodiae Fructus and its purified fructans (MOP70-1, MOP70-2, and MOW90-1) can appreciably promote MC3T3-E1 cells proliferation, differentiation, and mineralization, among which MOP70-2 and MOW90-1 can upregulate the marker genes expression of osteogenic differentiation, including Runx2, OSX, OC, OPN, BSP, and OPG, to stimulate osteoblast differentiation (Jiang et al., 2018; Yan et al., 2019). Through OVX and MC3T3-E1 cell models, Zhang et al. demonstrated that monotropein (40 and 80 mg·kg-1·day-1) can increase bone mineral content (BMC), BMD, BVF, improve bone microstructure, and enhance maximum stress and elastic modulus. In addition, monotropein can lower the levels of serum IL-1, IL-6, and sRANKL in OVX rats (Zhang N et al., 2016).

Taken together, some important KTTCMs, such as Drynariae Rhizoma, Epimedii Folium, and Psoraleae Fructus, contain various active chemical components, including flavonoids, saponin, lignans, and coumarins. They are prone to act synergistically to exhibit improved anti-osteoporotic effects than the single compound; this will provide a solid foundation for further research.

## Clinical efficacy of kidney tonifying traditional Chinese medicine therapy for osteoporosis

Compared with chemical drugs, KTTCM possesses unique advantages in the treatment of osteoporosis by influencing the fate of bone cells during bone remodeling. It is characterized by focusing on the overall regulation and dialectical treatment and can be used as an ideal alternative medicine for osteoporosis.

Masses of medical practice and meta-analysis indicate that KTTCM can not only improve bone quality and biomechanical properties but also alleviate back pain, lumbar debility, and other symptoms.

The classic KTTCM recorded in ancient medicine books have anti-osteoporosis efficacy, such as Qing’e pill, Zuogui pill, and Yougui pill. They are used to treat osteoporosis by reinforcing the kidney. In addition, TCM physicians could generally adjust one or more botanical drugs according to the differentiation of the patient’s physical condition and change their dosage in the prescription (Zhang Z et al., 2016). The recorded books, ingredients, clinical results, and references to classic formulas for the prevention and treatment of OP are summarized in Table 2.

**TABLE 2:** Ingredients of Classic formulas for the therapy of osteoporosis.

Formula	Recorded books	Ingredients/1000 preparation unit (aqueous extract)	Clinical results	Reference
Xianling Gubao capsule	Pharmacopoeia of the People’s Republic of China	Epimedium brevicornu Maxim. [Berberidaceae; Epimedii Folium] (1167 g), Dipsacus asper Wall. [Dipsacaceae; Dipsaci Radix] (167 g), Anemarrhena asphodeloides Bunge [Liliaceae; Anemarrhenae Rhizoma] (83 g), Salvia miltiorrhiza Bunge [Lamiaceae; Salviae miltiorrhizae Radix et Rhizoma] (83 g), Rehmannia glutinosa Libosch [Scrophulariaceae; Rehmanniae Radix] (83 g), and *Psoralea* corylifolia L. [Fabaceae; Psoraleae Fructus] (83 g)	Xianling Gubao capsule combined with calcium can improve femoral neck BMD and increase BGP and ALP contents in serum	Hui et al. (2022)
Gushukang capsule	Pharmacopoeia of the People’s Republic of China	Epimedium brevicornu Maxim. [Berberidaceae; Epimedii Folium] (17.5 g), Rehmannia glutinosa Libosch [Scrophulariaceae; Rehmanniae Radix] (23.2 g), Salvia miltiorrhiza Bunge [Lamiaceae; Salviae miltiorrhizae Radix et Rhizoma] (11.6 g), Drynaria fortunei (Kunze ex Mett.) J. Sm. [Polypodiaceae; Drynariae Rhizoma] (11.6 g), *Astragalus* membranaceus (Fisch.) Bunge var.mongholicus (Bunge) P. K. Hsiao [Fabaceae; Astragali Radix] (17.5 g), Auricularia auricula (L. ex Hook.) Underwood [Auriculariales; Auricularia Calvatia] (9.3 g), and Cucumis sativus L. [Cucurbitaceae; Cucumis Feuctus] (9.3 g)	Gu Shu Kang capsule can improve the level of BMD, BGP, ALP, and E2 and regulate urinary calcium creatinine ratio, serum calcium, and serum phosphorus	Guangwei et al. (2020)
Gusongbao granules	Pharmacopoeia of the People’s Republic of China	Epimedium brevicornu Maxim. [Berberidaceae; Epimedii Folium] (650 g), *Ostrea* gigas Thunberg [Osteroida; Ostreae Concha] (10 g), Dipsacus asper Wall. [Dipsacaceae; Dipsaci Radix] (50 g), Rehmannia glutinosa Libosch [Scrophulariaceae; Rehmanniae Radix] (40 g), Anemarrhena asphodeloides Bunge [Liliaceae; Anemarrhenae Rhizoma] (50 g), Paeonia lactiflora Pall. [Asclepiadaceae; Paeoniae Radix Alba] (50 g), Ligusticum chuanxiong Hort. [Apiaceae; chuanxiong Rhizoma] (50 g), Sparganium stoloniferum (Graebn.) Buch.-Ham. ex Juz. [Sparganiaceae; Spargani Rhizoma] (50 g), and Curcuma phaeocaulis Valeton [Zingiberaceae; Curcumae Rhizoma] (50 g)	Gusongbao granules combined with elcatonin can improve bone conversion factors and inflammatory cytokines and can also reduce ODI score	Li L et al. (2021)
Hugu capsule	Pharmacopoeia of the People’s Republic of China	Polygonum multiflora Thunb. [Polygonaceae; Polygoni Multiflori Rhizoma] (347.5 g), Epimedium brevicornu Maxim. [Berberidaceae; Epimedii Folium] (277.5 g), Rehmannia glutinosa Libosch [Scrophulariaceae; Rehmanniae Radix] (347.5 g), Chinemys reevesii (Gray) [Emydidae; Testudinis Carapax et Plastrum] (208.5 g), *Morinda* officinalis How [Rubiaceae Juss; Morindae Officinalis Radix] (277.5 g), Eucommia ulmoides Oliv. [Eucommiaceae; Eucommia Cortex] (277.5 g), Dipsacus asper Wall. [Dipsacaceae; Dipsaci Radix] (277.5 g), Drynaria fortunei (Kunze ex Mett.) J. Sm. [Polypodiaceae; Drynariae Rhizoma] (277.5 g), Angelica sinensis (Oliv.) Diels [Apiaceae; Angelicae Sinensis Radix] (277.5 g), and *Dioscorea* opposita Thunb. [Dioscoreaceae; Dioscoreae Rhizoma] (400 g)	Hugu capsule can increase BMD, reduce body pain, and improve the quality of life of patients	Yang et al. (2019)
Jintiange capsule	Pharmacopoeia of the People’s Republic of China	Panthera tigris L. [Factitial os tigris] (400 g)	Jintiange capsule can improve the clinical efficacy rate, vertebral height, Cobb’s angle, bone mineral density, pain relief, and daily activity function	Shu and Zhang (2022)
Qianggu capsule	Pharmacopoeia of the People’s Republic of China	Total flavonoids of Drynaria fortunei (Kunze ex Mett.) J. Sm. [Polypodiaceae; Drynariae Rhizoma] (400 g)	Qianggu capsule combined with Caltrate D is better than Caltrate D on lumbar spine BMD, femoral neck BMD, and femoral great trochanter BMD.	Wei et al. (2017)
Qing’e pill	Tai Ping Hui Min He Ji Ju Fang	*Psoralea* corylifolia L. [Fabaceae; Psoraleae Fructus] (480 g), Eucommia ulmoides Oliv. [Eucommiaceae; Eucommia Cortex] (240 g), Juglans regia L. [Juglandaceae; Juglandis Semen] (150 g), and Allium sativum L. [Liliaceae Juss.; Allii Sativi Bulbus] (120 g)	Qing’e pill + conventional treatment can relieve patients’ osteoporosis pain and improve femoral word BMD, femoral neck BMD, and femoral greater trochanteric BMD.	Chen et al. (2021)
Yishen Zhuanggu decoction	Self-made	Drynaria fortunei (Kunze ex Mett.) J. Sm. [Polypodiaceae; Drynariae Rhizoma] (300 g), *Cyperus* rotundus L. [Cyperaceae; Cyperi Rhizoma] (200 g), Cynomorium songaricum Rupr. [Cynomoriaceae; Cynomorii Herba] (150 g), and Cervi Cornus Colla (150 g)	Yishen Jiangu decoction can restore the height of the vertebral body, stabilize the lumbar spine, relieve pain, improve the function of the lumbar spine, and improve the quality of life	Xue L et al. (2022)
Bushen Jianpi Zhuanggu formula	Self-made	Drynaria fortunei (Kunze ex Mett.) J. Sm. [Polypodiaceae; Drynariae Rhizoma] (100 g), Dipsacus asper Wall. [Dipsacaceae; Dipsaci Radix] (50 g), *Psoralea* corylifolia L. [Fabaceae; Psoraleae Fructus] (100 g), Lycium barbarum L. [Solanaceae; Lycii Cortex] (150 g), Eucommia ulmoides Oliv. [Eucommiaceae; Eucommia Cortex] (50 g), Euryale ferox Salisb. [Nymphaeaceae; Euryales Semen] (100 g), Cibotium barometz (L.) J. Sm. [Dicksoniaceae; Cibotii Radix] (200 g), and *Canis familiaris* Linnaeus [Canidae; Canise Ossis] (50 g)	Bushen Jianpi Zhuanggu formula can increase the levels of BGP, PINP, ALP, beta-CTX, and NTX.	Chen F et al. (2022)
Zuogui pill	Jing Yue Quan Shu	Rehmannia glutinosa Libosch [Scrophulariaceae; Rehmanniae Radix] (240 g), *Dioscorea* opposite Thunb. [Dioscoreaceae; Dioscoreae Opposicae Rhizoma] (120 g), Lycium barbarum L. [Solanaceae; Lycii Cortex] (120 g), Cornus officinalis Sieb. Et Zucc. [Cornaceae; Corni Fructus] (120 g), Cyathula officinalis Kuan [Amaranthaceae; Cyathulae Radix] (90 g), Cuscuta chinensis Lam. [Convolvulaceae; Cuscutae Semen] (120 g), *Cervus elaphus* Linnaeus [Cervidae; Cervi Cornu] (120 g), and Chinemys reevesii Gray [Emydidae; Testudinis Carapax et Plastrum] (120 g)	Zuogui pill can improve the levels of ALP, BGP, NBAP, and TRAP.	Li S et al. (2021)
Yougui pill	Jing Yue Quan Shu	Rehmannia glutinosa Libosch [Scrophulariaceae; Rehmanniae Radix] (240 g), Aconitum carmichaeli Debx. [Ranunculaceae; Aconiti Radix] (120 g), Cinnamomum cassia Presl [Lauraceae; Cinnamomi Cortex] (120 g), *Dioscorea* opposita Thunb. [Dioscoreaceae; Dioscoreae Rhizoma] (120 g), Cornus officinalis Sieb. Et Zucc. [Cornaceae; Corni Fructus] (120 g), Cuscuta chinensis Lam. [Convolvulaceae; Cuscutae Semen] (120 g), *Cervus elaphus* Linnaeus [Cervidae; Cervi Cornu] (90 g), Lycium barbarum L. [Solanaceae; Lycii Cortex] (90 g), Angelica sinensis (Oliv.) Diels [Apiaceae; Angelicae sinensis Radix] (60 g), and Eucommia ulmoides Oliv. [Eucommiaceae; Eucommia Cortex] (60 g)	Yougui pill can improve BMD, ease pain, relieve kidney deficiency syndrome, improve the quality of life of osteoporosis patients, inhibit bone conversion, and regulate the coupling balance of bone formation and bone resorption	Li et al. (2018)

Traditionally, TCM formulas are more extensively applied than single botanical drugs in the treatment of OP. Different botanical drugs existing in TCM formulas for the therapy of osteoporosis usually contain active compounds (Gao et al., 2021). In addition, several researchers have suggested that combining two or more natural products may enhance multiple mechanisms of action and present synergistic effects and fewer side effects than treating with one natural product alone (Park et al., 2020). Aconiti Lateralis Radix Praeparaia is a Chinese herbal medicine, which possesses the effects of cardiac failure, diarrhea, arthralgia, and edema, but is toxic, and it is frequently combined with Zingiberis Rhizoma to increase efficacy and reduce toxicity (Peng et al., 2013). The Chinese herb pair of Aconiti Lateralis Radix Praeparaia and Zingiberis Rhizoma is a typical TCM combination that is commonly used in the clinic, and it has anti-neuroinflammatory effects and can effectively alleviate tumor-associated fatigue and its depressive symptoms, which is more effective than the two herbal medicines alone (Yang et al., 2021). In addition, the botanical drug combination of Angelica sinensis Radix and Carthami Flos is commonly used to treat blood stasis syndrome (Yue et al., 2017), and the botanical drug couple Salviae miltiorrhizae Radix et Rhizoma and Puerariae Thomsonii Radix is used for the treatment of osteoporosis (Qin et al., 2020), and their effectiveness is superior to that of one natural product alone.

Therefore, identifying the active compounds in TCM formulas and determining their ingredients can help identify safe drug candidates and synergistic agents in the process of drug discovery.

### Anti-osteoporosis mechanisms of kidney tonifying traditional Chinese medicine

**FIGURE 3:**
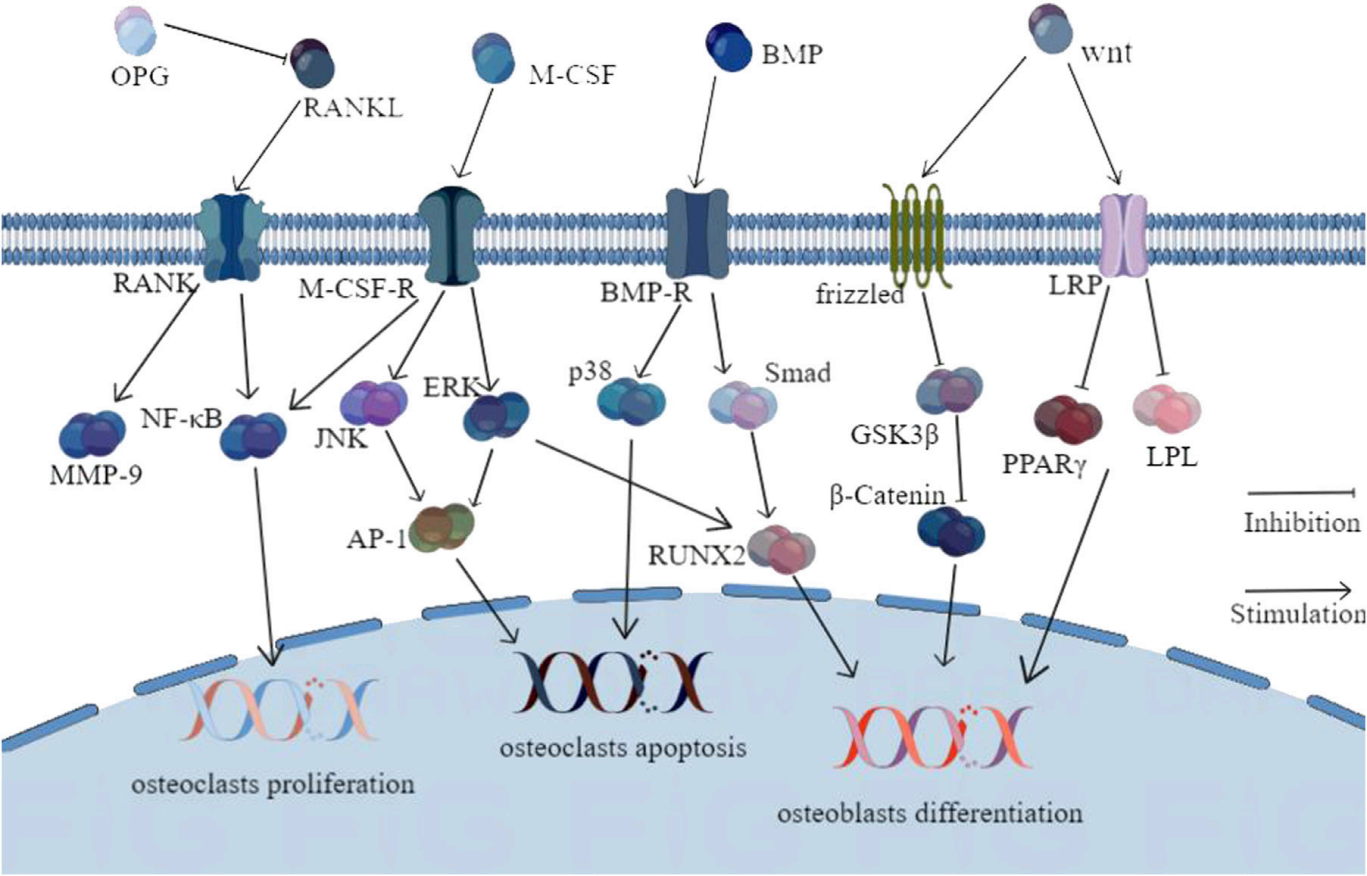
Molecular pathways involved in the anti-osteoporosis actions of KTTCM.

Over the last 2 decades, studies revealed that the primary cause of osteoporosis is bone metabolism disorder, which is primarily determined by the imbalance between bone formation and bone resorption (Park et al., 2019). Many investigations recommend that bone metabolism involves a series of complex factors, including molecular and cellular events, such as apoptosis, inflammatory response, oxidative stress, and autophagy (Gatti et al., 2020). Those events are regulated by multiple pathways that are the fundamental of anti-osteoporosis effect from KTTCM, including the OPG/RANKL/RANK, BMP/Smads, MAPKs, and Wnt/β-catenin pathways (Figure 3)

### OPG/RANKL/RANK signaling pathway

The RANKL-OPG relationship is one of the most crucial pathways in bone resorption and bone formation. RANKL can bind to RANK on the osteoclast and its precursors and then induce the differentiation, fusion, and activation of osteoclast. OPG can bind to RANKL to prevent RANKL–RANK interaction to inhibit bone resorption. KTTCM can adjust the ratio of RANKL/OPG to inhibit bone resorption. For example, icariin (10–8 M) can regulate the gene expression of OPG and RANKL and inhibit LPS-induced bone resorption by inhibiting IL-6 and TNF-α and suppressing hypoxia-inducible factor-1α (HIF-1α) (Hsieh et al., 2011). Ikarisoside A (5 and 10 × 10–6 M) also can inhibit the RANKL-mediated activation of JNK, NF-κB, and Akt, downregulate the expression of MMP-9, TRAP, RANK, and CTSK, and inhibit OC formation in mouse RAW 264.7 cells (Choi et al., 2010). TFDR can dose-dependently upregulate OPG and estradiol expression and downregulate RANK and RANKL expression in OP model rats, thereby regulating the OPG/RANKL/RANK axis (Liu et al., 2015). Corni Fructus (100 μg·mL-1) can significantly inhibit the RANKL-mediated formation of osteoclast and the differentiation of bone marrow-derived macrophages (BMMs), and its mechanism is associated to suppress the protein expression of c-Fos and NFATc1 and inhibit the phosphorylation of p38 and JNK induced by RANKL (Kim et al., 2012).

### MAPK signaling pathway

MAPK belongs to the secondary messenger family which is responsible for transmitting various extracellular signals into the cells, and it is involved in complex cellular processes like proliferation, differentiation, transformation, and apoptosis. It plays an important role in cellular responses to growth factors, cytokines, or environmental stress (Yang et al., 2013; Chiranjib et al., 2016). Three main pathways are related to MAPK and OP prevention and treatment: ERK, p38MAPK, and JNK pathways, which mainly improve bone metabolism by regulating the activation, proliferation, and differentiation of OB and OC (Kim et al., 2013). Zhu et al. investigated the influence of TFDR on the phosphorylation of ERK1/2 and p38 in SD rats under the action of advanced glycation end products (AGEs). The results showed that TFDR (50 μg·mL-1) could dose-dependently increase the expression and mineralization capacity of ALP, Col I, and BGP in OB, stimulate the phosphorylation of ERK1/2 and p38, and prevent and treat OP(Xiaofeng et al., 2013). Icariin (10–8 M) can inhibit the proliferation and differentiation of OC by inhibiting p38 and JNK activation in the co-culture system of osteoblasts and BMSCs induced by LPS, suggesting that icariin can inhibit LPS-induced activation of osteoclast by p38 and JNK pathway (Hsieh et al., 2011).

### BMP/Smads signaling pathway

BMP, a member of the transforming growth factor-beta (TGF-β) superfamily, is highly associated with the differentiation and proliferation of OB. Runx2 is vital for OB differentiation as a crucial transcription factor. Interestingly, BMP binds heterodimeric receptors to stimulate Smad proteins, which activate osteoblastogenic genes *via* Runx2 (Lin Grace and Hankenson Kurt, 2011). Modern pharmacological researchers have found that Epimedii Folium exerts anti-osteoporosis effects through the BMP/Smads pathway. For example, icariin (10–5 M) can facilitate the osteogenesis of MC3T3-E1 cells by upregulating the expression of Runx2, increasing the levels of BMP-4, and activating the BMP signaling pathway (Jiyuan et al., 2008). In addition, maohuoside A (8 × 10–2 M) could activate Smad4 expression and enhance osteogenesis in mouse BMSCs osteoblasts *via* the BMP pathway (Ming et al., 2013). The aqueous extract of Cuscutae Semen (50 and 100 mg·mL-1) can dose-dependently increase the expression of BMP-2, Col, and ALP in MG-63 cells and promote the osteogenic activity of MG-63 cells (Mo et al., 2009). Psoralen can increase the expression of BMP-2/4 and phospho-Smad1/5/8 and induce the activation of BMP-related genes like 12xsbe-OC-Luc and ostrix, thereby promoting the osteogenic differentiation of OB (Yali et al., 2020). Asperosaponin Ⅵ (10–6 M) may promote MC3T3-E1 cells and primary osteoblasts proliferation, differentiation, and mineralization to increase bone formation by increasing ALP activity and increasing BMP-2 synthesis (Niu et al., 2011).

### Wnt/β-catenin pathway

The Wnt/β-catenin signaling pathway plays an essential role in bone metabolism, and it further controls bone growth. Wnt is a member of the highly conserved secreted glycoprotein family that binds with frizzled (FZD) and LDL receptor-related proteins (LRPs) to promote glycogen synthase kinase 3β (GSK3β) phosphorylation and inactivation and prevents β-catenin degradation. Furthermore, wnt transfers to the nucleus to induce the transcription of osteoblastic gene *via* forming a complex with a T-cell factor (Lin Grace and Hankenson Kurt, 2011). The study found that Drynariae Rhizoma decoction (6 g·kg-1·day-1) could inhibit adipogenic differentiation of BMSCs in OVX rats by inhibiting PPARγ and LPL mRNA expression, regulating Wnt/β-catenin signaling pathway, reducing adipocyte number and volume in bone marrow cavity, and increasing bone density (LI et al., 2019). In addition, total flavones from Cuscutae Semen (60, 120, and 180 mg·kg-1·day-1) can increase BMD and improve symptoms of osteoporosis by increasing the levels of β-catenin and OPG in OVX rats, reducing the levels of RANKL and DKK1, and regulating the Wnt/β-catenin signaling pathway (Zhao et al., 2018). Moreover, icariin (50 mg·mL-1) increases the expression of Runx2, p-Smad5, BMP-2, BMP-4, Wnt1, and β-catenin in BMSCs. This implies that icariin can promote the viability and osteogenic differentiation of BMSC by regulating BMP-2/Smad5/Runx2 and WNT/β-catenin pathways (Zhang X et al., 2021).

### Current status of pharmacological research on osteoporosis

In modern pharmacology research, the BMSCs, MG-63, and MC3T3-E1 cells were used to construct *in vitro* models, and the anti-osteoporosis activity of KTTCM was explored by examining indicators such as OPG, RANKL, ALP, and TRAP. Simultaneously, to understand the pathogenesis of osteoporosis and to evaluate drugs preclinically, three animal models, namely, castrated osteoporotic models, chemical drugs caused osteoporotic models, and disuse osteoporotic models, have been developed (Egermann et al., 2005). Previous studies on the anti-osteoporosis of KTTCM mostly use castrated osteoporotic models and lack other osteoporotic animals, such as osteoporotic models induced by glucocorticoids and disuse osteoporotic models. It is true that the castrated osteoporotic models can replicate postmenopausal cancellous bone loss relatively quickly; however, there is no Haversian system in cortical bone, no impaired osteoblast function during the late stages of estrogen deficiency, and no multicellular unit-based remodeling in the late stages of estrogen deficiency also can create a confounding effect on the results of measurements (Yousefzadeh et al., 2020). In addition, the biochemical indexes in cell tests cannot reflect the action characteristics of KTTCM. Therefore, some novel animal models, like disuse osteoporotic models, should be applied to study the anti-osteoporosis of KTTCM. In addition, the cell and animal models should be combined to select relevant parameters to highlight the anti-osteoporosis properties of KTTCM.

### Safety and toxicity

With their efficacy and safety, TCM is returning to a vital position in the field of health. A case in point is that the active component of Alpiniae officinarum Rhizoma, galangin, can inhibit the proliferation of human laryngeal cancer cell lines (tu212, m4e, and Hep-2) while causing no toxic effects to normal cells (HBE, hhl-5, and RTE) through p38 and Akt/NF-κB/mTOR pathway, exhibiting great anti-laryngeal cancer effects and security (Wang and Tang, 2017). As an attractive anti-cancer drug therapy, the combination of curcumin (CUR) and paclitaxel (PTX) can significantly inhibit metastasis of lung cancer and breast cancer. Specifically, an IC50 of 13.24 μg·mL-1 of Cur-Ptx was observed against A549 cells, and only 1.450 μg·mL-1 of Cur-Ptx was required to attain IC50 in MDA-MB-231 cells, which is less than using Ptx alone. Remarkably, at least 2- and 26-fold higher concentrations of Cur-PTX were required to cause MRC-5 cytotoxicity compared with the doses required for A549 and MDA-MB-231 cells (Muthoosamy et al., 2016). This plainly reveals that the safety of TCM is higher than that of current mainstream clinical chemotherapeutic agents.

In recent years, the frequent adverse reactions of TCM not only affected the safety of drug use but also seriously hindered the development and internationalization of TCM. For instance, TCM is effective in treating COVID-19, but it is still questioned by the international community about the safety problems in TCM therapy (Cyranoski, 2020). KTTCM has excellent anti-osteoporosis effect, but its safety and toxicity are still two concerns in clinical application, and the underlying mechanisms of KTTCM-induced toxicity remain unclear. To further evaluate the potential risks of KTTCM and guide its safe use, some studies on the nephrotoxicity, hepatotoxicity, and embryotoxicity of KTTCM are summarized in the following section.

### Liver damage

Hepatotoxicity refers to the damage caused to the liver by the drug or its metabolites during the administration of medicine (Shen et al., 2019). The liver is the main site of the metabolism of substances, so hepatotoxicity of drugs is a relatively common toxic side effect (Hoofnagle and Björnsson, 2019). Zhang et al. analyzed the components extracted from Epimedii Folium by UPLC-Q-TOF-MS and evaluated the cytotoxicity of 2″-O-Rhamnosyl icariside II (66 μg·mL-1), baohuoside I (32 μg·mL-1), and baohuoside II (36 μg·mL-1) on HL-7702 and HepG2 cells. The result showed Epimedii Folium extract affected the leakage of alanine aminotransferase (ALT), aspartate aminotransferase (AST), and lactate dehydrogenase (LDH), decreased SOD, GSH-Px, and MMP activities, and increased MDA activity and intercellular reactive oxygen species (ROS) levels. The mechanism of hepatotoxicity may be associated with damaged cell structure, increased oxidative stress, and induction of apoptosis (Hoofnagle and Björnsson, 2019; Zhang et al., 2020). Yu et al. found that psoralen (14 mg·kg-1) caused disorders in multiple amino acid metabolic pathways in rats, leading to liver damage (Yu et al., 2019). Zhou et al. showed a hepatocyte cell cycle arrest that can be caused by psoralen (400 and 800 mg·kg-1·day-1), resulting in decreased liver regeneration and self-healing capacity. The mechanism involves the inhibition of the mTOR signaling pathway, which is implicated in mitochondrial damage, reduced liver regeneration, and inducing liver injury (Zhou et al., 2018).

### Renal damage

The kidney is an important organ responsible for the excretion of various toxic metabolic waste products, which has various physiological functions such as absorption, distribution, metabolism, and excretion, and the risk of the kidney contacting the toxic components is greatly increased (Oh, 2020). Xu et al. established a serum metabolomic method based on UPLC-Q-TOF-MS to identify ten endogenous metabolites from drug-containing serum and evaluate the toxicity of osteoclast through hematological indicators, biochemical indicators, and histological changes. The results showed that long-term administration of the ethanolic extract of Psoraleae Fructus (1.62, 1.08, and 0.54 g·kg-1·day-1) could cause liver and kidney injury in rats (Xu Y et al., 2021). A study has shown that isopsoralen (60 mg·kg-1·day-1) has certain nephrotoxicity, which can lead to a decrease in body weight of rats, an increase in kidney coefficient, a significant increase in serum urea nitrogen content, and vacuolar degeneration in the distal convoluted tubule cortex of the rat kidney (Zhang Y et al., 2019). The mechanism correlates with the downregulation of mRNA of transcripts such as mOCT1, mOCTN1, and mOAT3 in the kidney, upregulation of mOAT1 expression, and decreased protein levels of mOCTN2, which leads to dysfunction of the renal organic ion transport system (Wang et al., 2012).

### Embryotoxicity

Embryotoxicity refers to the damage caused to the embryo and fetus by exogenous drugs and encompasses four aspects: death, growth retardation, malformations, and functional defects (Barrow, 2016). A passive diffusion study of psoralen (10–4 M) in the BeWo cell line demonstrated that it crossed the placental barrier and was well absorbed. Consequently, psoralen may be harmful to pregnant women as it causes embryotoxicity (Jie et al., 2015). Some studies have indicated that Cuscutae Semen aqueous extract (40 g·kg-1·day-1) has potential genotoxicity as its extract can enhance the micronucleus rate of bone marrow cells and embryonic liver cells of pregnant mice and induce the increase of micronucleus rates in pregnant mice and embryos (Xia and Li, 2014). Furthermore, the drug-containing serum of Cuscutae Semen (10 g·kg-1·day-1) could inhibit the outgrowth of forelimb buds in SD rat embryos (Liu H et al., 2020). Liu et al. found that Dipsaci Radix aqueous extract (32 g·kg-1·day-1) is genotoxic, and it can induce an increase in the micronucleus rate of bone marrow eosinophilic erythrocytes of mice (Liu et al., 2016).

## Conclusion

OP has a large impact on the general health of those aged over 60 years, and it can increase fracture risk, which further results in decreased quality of life, disability, institutionalization, and even excessive mortality, putting a heavy burden on the health system (Surakka et al., 2020). When OP patients enter their active phase, they usually exhibit characteristic symptoms, such as lumbar debility and bone pain. As society is aging, the prevention and treatment of OP is a particularly significant puzzle in the medical system (Osnes et al., 2004). KTTCM can regulate the balanced activation of OB and OC, increase the level of estrogen, regulate the osteogenic and adipogenic differentiation of MSCs, resist oxidative stress, and promote calcium and phosphorus metabolism through OPG/RANKL/RANK, BMP/Smads, MAPKs, and Wnt/β-catenin (Zhang N et al., 2016). In addition, some research studies have shown that KTTCM has serious adverse reactions, including nephrotoxicity, hepatotoxicity, and embryotoxicity. In traditional Chinese medicine, multiple ingredients, multiple targets, and multiple pathways work synergistically to produce their efficacy (Sirui et al., 2019). Compared with the clinical drugs commonly used for anti-osteoporosis, KTTCM exhibited great potential for therapeutic translation as a simple and effective treatment of OP, with improved drug efficacy and minimal toxicity. These data demonstrate that KTTCM can be a promising potential candidate for therapeutic use in OP. Consequently, kidney tonifying traditional Chinese medicine needs further in-depth research because it might provide an alternative therapeutic option for OP.

Safety is the prime concern during the entire cycle of drug development and application. KTTCM has a significant anti-osteoporosis effect, whereas some TCM has been documented to have nephrotoxicity, hepatotoxicity, and embryotoxicity, which has been questioned for its ambiguous composition and toxicology, and the relative relationship between the medicinal components and the toxic components remains unclear. For example, psoralen and isopsoralen are not only effective components but also toxic components of Psoraleae Fructus (Yali et al., 2020). It is of conspicuous significance to study the toxic components of kidney tonifying traditional Chinese medicine and its dose–effect–toxicity relationship for the development of anti-osteoporosis drugs.

Further investigations of kidney tonifying traditional Chinese medicine were proposed to examine the effects and mechanisms of the pharmacodynamic components in a more detailed way. On the one hand, the anti-osteoporosis activity of some active ingredients such as icariin, naringin, and psoralen has been verified in experiments performed in suitable animal models. Nevertheless, TCM extracts are an effective aggregation of multiple components, and a single component cannot completely reveal its pharmacological activity. On the other hand, TCM extracts have a good effect on the prevention and treatment of OP, but the components are complex and changeable, and the effectiveness of single or multicomponent interventions, however, is not clearly delineated. Notably, most of the anti-osteoporosis mechanism trials are based on a single signaling pathway, and the mechanism of action of TCM is not a single pathway, but it is multirooted and multitargeted. Moreover, in the absence of any robust data on medication-related clinical information, kidney tonifying traditional Chinese medicine is difficult to be widely used, and its anti-osteoporosis effect is difficult to verify. Accordingly, the subsequent research can present a pharmacological activity-guided profiling strategy to characterize the chemical components in kidney tonifying traditional Chinese medicine in multiple directions and at multiple levels *via* using biological affinity chromatography and many omics technologies. Although the anti-osteoporosis effects of kidney tonifying traditional Chinese medicine are well studied, more relative clinical trials are still needed for further investigation, not just for exploring the anti-osteoporosis mechanism of kidney tonifying traditional Chinese medicine through the metabolic process of each component in the body. Equally, efforts should be put to conduct large-scale controlled clinical studies on TCM with a long history of medicinal use and definite clinical curative effect combined with the dialectical thought of Chinese medicine, the medication holistic view, and modern medicine efficacy research.
